# A Case of a Ruptured Microaneurysm at the Tip of the Basilar Artery With Right Abducens Nerve Palsy at the Time of the Initial Rupture and Rerupture During an Outpatient Follow-Up

**DOI:** 10.7759/cureus.31797

**Published:** 2022-11-22

**Authors:** Seigo Kimura, Ryokichi Yagi, Keiichi Yamada, Hirokatsu Taniguchi, Masahiko Wanibuchi

**Affiliations:** 1 Department of Neurosurgery, Kouzenkai Yagi Neurosurgical Hospital, Osaka, JPN; 2 Department of Neurosurgery, Osaka Medical and Pharmaceutical University, Takatsuki, JPN

**Keywords:** tip of basilar artery, aneurysmal subarachnoid hemorrhage, stent-assisted coil embolization, regrowth, abducens nerve palsy

## Abstract

The presentation of abducens nerve palsy after each occurrence of subarachnoid hemorrhage (SAH) is rare. Herein, we report the case of a patient with a ruptured microaneurysm at the tip of the basilar artery who presented with right abducens nerve palsy at the time of the initial rupture and rerupture during an outpatient follow-up. A 52-year-old woman developed SAH with right abducens nerve palsy, which was treated with coil embolization. One year after the initial SAH, there was a relapse of the SAH and paresis of the right abducent nerve palsy. This may have been caused by the location of the abducens nerve in relation to the surrounding structures, which were susceptible to the effects of hematoma or intracranial pressure fluctuations. Stent-assisted coil embolization is an effective treatment for regrowth that appears after endovascular therapy of microaneurysms.

## Introduction

Subarachnoid hemorrhage (SAH) with abducens nerve palsy has been reported in several studies [1-4]; however, its frequency, mechanism of occurrence, and clinical course remain unclear. No cases of ruptured cerebral aneurysms with abducens nerve palsy on the same side have yet been reported at the time of the initial rupture and rerupture during an outpatient follow-up. Herein, we report the case of a patient with a ruptured microaneurysm at the tip of the basilar artery who was presented with a right abducens nerve palsy at the time of the initial rupture and rerupture during an outpatient follow-up.

## Case presentation

**Figure 1:**
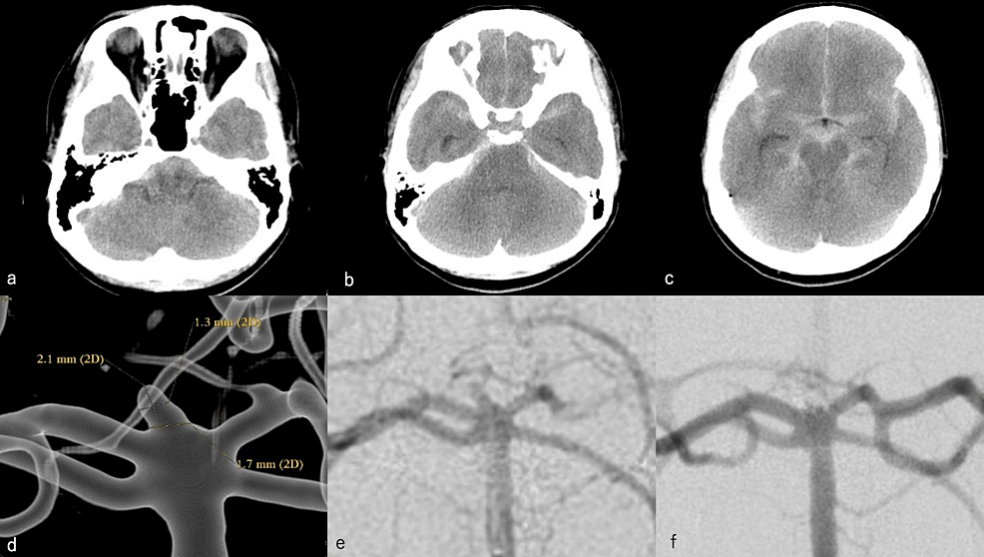
Coil embolization for the first ruptured aneurysm. (a)-(c). Head CT revealed SAH (Fisher group 3) with a WFNS grade of II and a Hunt & Kosnik grade of II. (d) DSA shows a microaneurysm with a longitudinal diameter of 2.1 mm at the tip of the basilar artery. (e) Left vertebral angiography. (f) The aneurysm was contrast-enhanced, and a 2 mm × 3 cm Target Nano was implanted. The aneurysm was no longer contrasted, and treatment was completed. SAH: subarachnoid hemorrhage; WFNS: World Federation of Neurosurgical Societies; CT: computed tomography; DSA: digital subtraction angiography.

On October 24, 2019, a 52-year-old woman was rushed to the emergency room because of the sudden onset of a severe headache. Her medical history revealed no significant findings. After admission to the emergency room, her Glasgow Coma Scale (GCS) score was 13 (E4, V5, and M4). She had no obvious pupillary irregularity but she was presented with tetraplegia and frequent vomiting. Her blood pressure level and pulse rate were 215/100 mmHg and 43 beats/min, respectively. Head computed tomography (CT) revealed SAH (Fisher group 3) with a World Federation of Neurosurgical Societies (WFNS) grade of II and a Hunt & Kosnik grade of II (Figure 1a-1c). Digital subtraction angiography (DSA) showed a microaneurysm with a longitudinal diameter of 2.1 mm at the tip of the basilar artery (Figure 1d); therefore, emergency coil embolization was performed. A 6-Fr Roadmaster guiding catheter (Goodman Co., Ltd., Aichi, Japan) was inserted into the left vertebral artery (VA), and a 3.4-Fr Tactics (Technocrat, Aichi, Japan) distal access catheter was used. An SL10 microcatheter (Stryker, Kalamazoo, MI, USA) was inserted into the aneurysm using a Traxcess 14 guidewire (Terumo Corporation, Tokyo, Japan) (Figure 1e). The aneurysm was contrast-enhanced, and a 2 mm × 3 cm Target Nano (Stryker, Kalamazoo, MI, USA) was implanted. The treatment was considered complete when the aneurysm was no longer contrasted (Figure 1f).

**Figure 2:**
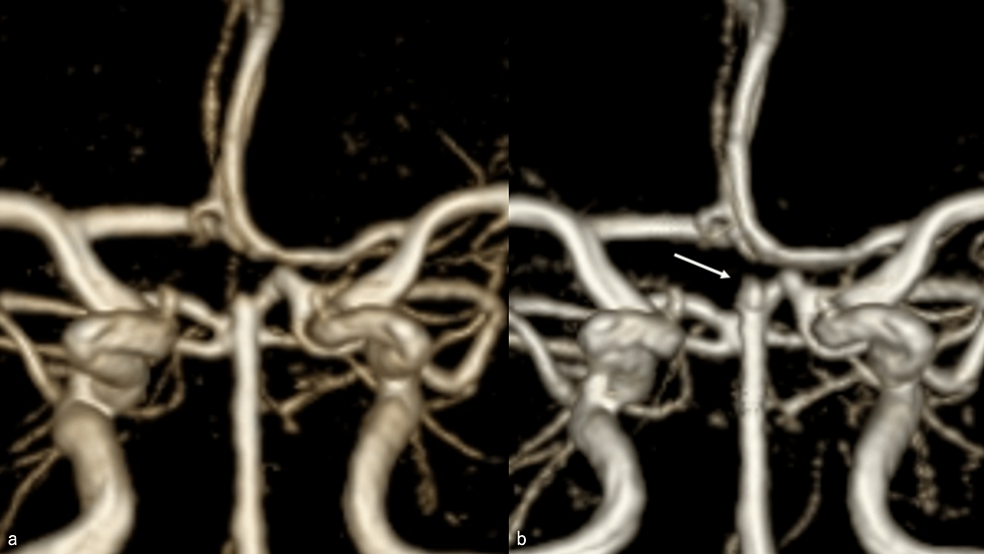
Head MRA six months and one year after surgery. (a) At six months after the disease onset, an outpatient MRA of the head revealed no change. (b) However, one year after the disease onset, findings suggestive of a recurrence of the cerebral aneurysm were noted (white arrow). MRA: magnetic resonance angiography.

When the patient was extubated the day after her surgery, she presented with diplopia and right abducens nerve palsy. However, her head magnetic resonance imaging (MRI) revealed no obvious acute cerebral infarction, and head magnetic resonance angiography (MRA) indicated no cerebral aneurysm or main trunk artery occlusion. Her right abducens nerve palsy gradually improved; moreover, she fully recovered on day 14, November 7, 2019, and was discharged on day 21, November 14, 2019, with a modified Rankin Scale (mRS) score of 0. In the postoperative course, fasudil hydrochloride hydrate, 90 mg/day, was administered, and no cerebral vasospasm was observed. At six months after the disease onset, an outpatient MRA of the head revealed no change (Figure 2a).

**Figure 3:**
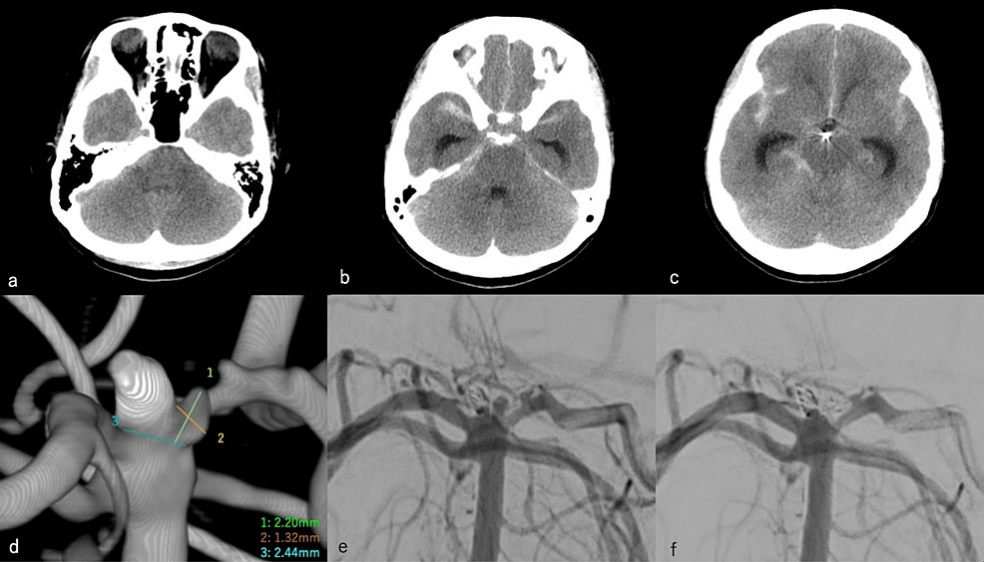
Coil embolization for the recurrence of cerebral aneurysm. (a)-(c): Head CT revealed subarachnoid hemorrhage (Fisher group 3), with a Hunt & Kosnik grade of Ⅲ and a WFNS grade of Ⅲ. (d) DSA revealed a recurrence with a length of 2.2 mm in the neck region of the initially treated cerebral aneurysm. (e) Left vertebral angiography. (f) A 1.5 mm × 2 cm Hyper Soft 3D was implanted, and embolization was performed. WFNS: World Federation of Neurosurgical Societies; CT: computed tomography; DSA: digital subtraction angiography.

However, one year and four months after the disease onset, on March 3, 2021, an outpatient MRA revealed findings suggestive of the regrowth of a cerebral aneurysm (Figure 2b). For the patient’s convenience, we planned to perform the examination on April 2, 2021; however, two days before the scheduled examination, on March 31, 2021, she was found crouching in the bathroom by her family at home and was rushed to the hospital for emergency treatment. After admission to the emergency room, her GCS score was 9 (E2, V2, and M5), without pupillary irregularity, and she did not have any gross neurological deficits. Her blood pressure level was 152/67 mmHg, and her pulse rate was 54 beats/min. Head CT revealed SAH (Fisher group 3), with a Hunt & Kosnik grade of III and a WFNS grade of III (Figure 3a-3c). DSA was immediately performed, which revealed regrowth with a length of 2.2 mm in the neck region of the initially treated cerebral aneurysm (Figure 3d).

**Figure 4:**
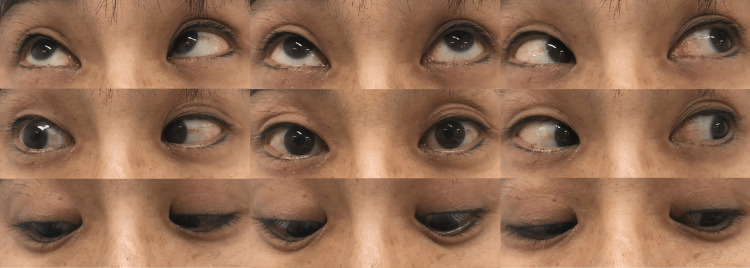
Patient's eye movements. The right abducens nerve palsy was still present on day 12, although it was improving.

After sedation, coil embolization was performed by the supervising neuroendovascular therapist on day 2, April 2, 2021. A 6-Fr Roadmaster catheter was guided to the left VA (Figure 3e), and Tactics and SL10 were guided to the area of regrowth of the cerebral aneurysm using Traxcess 14. A 1.5 mm × 2 cm Hyper Soft 3D (MicroVention/Terumo, CA, USA) was implanted, and embolization was performed (Figure 3f). When the patient was extubated on day 3, April 3, 2021, she had diplopia and right abducens nerve palsy. She did not have any gross neurological deficits. A diffusion-weighted image of the head MRI revealed no high signal intensity area, and the head MRA showed no cerebral aneurysm or obvious main artery occlusion. The right abducens nerve palsy was still present on day 12, April 12, 2021, although it was improving (Figure 4). Thereafter, although subjective symptoms of diplopia persisted, the right abducens nerve palsy gradually improved, and the patient was discharged from the hospital on day 30, April 30, 2021, with an mRS score of 0. In the postoperative course, fasudil hydrochloride hydrate, 90 mg/day, was administered, and no cerebral vasospasm was observed.

**Figure 5:**
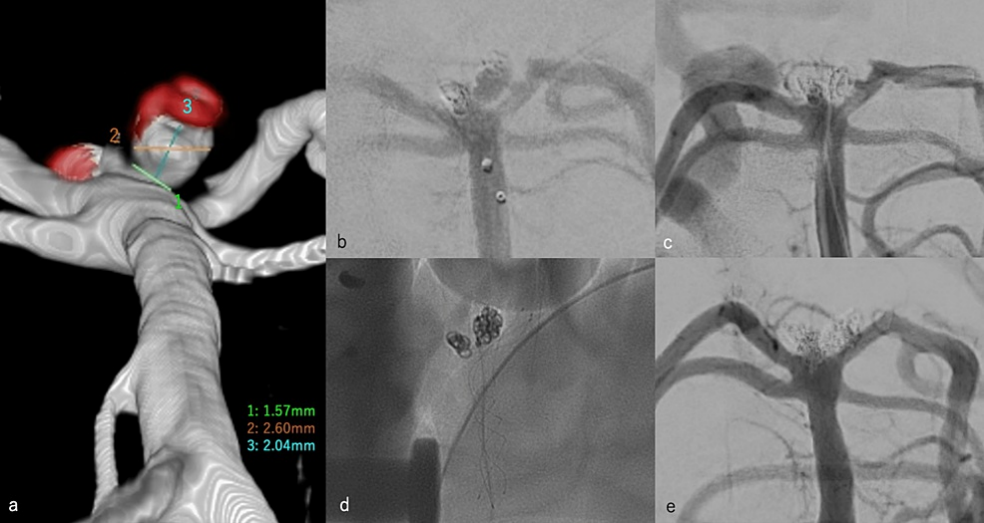
Stent-assisted coil embolization for the second recurrence of a cerebral aneurysm. (a) DSA revealed the second regrowth of the cerebral aneurysm. (b) A 6-Fr Roadmaster catheter was guided to the left VA, and Headway 17 and SL10 were guided to the basilar artery. (c) Headway 17 was guided to the left posterior cerebral artery, whereas SL10 was guided to the second regrowth of the cerebral aneurysm. (d) A 3.5 mm × 23 mm LVIS Jr. was deployed. (e) A 1.5 mm × 3 cm HyperSoft 3D was implanted through the jail lumen of the stent; then, the coil diameter was lowered, and six coils were implanted. DSA: digital subtraction angiography; VA: vertebral artery.

During an outpatient examination, two months after the rerupture, during an outpatient follow-up on March 29, 2021, the right abducens nerve palsy had completely improved; however, an outpatient MRA of the head on the same day revealed findings suggestive of cerebral aneurysm re-enlargement. Therefore, a DSA was performed on June 4, 2021, which confirmed the second regrowth of the cerebral aneurysm (Figure 5a). She was then administered 75 mg of clopidogrel and 100 mg of aspirin (dual antiplatelet therapy (DAPT)), and stent-assisted coil embolization was performed on June 14, 2021. A 6-Fr Roadmaster catheter was guided to the left VA, and Headway 17 (MicroVention, Terumo, Tustin, CA, USA) and SL10 were guided to the basilar artery (Figure 5b). Subsequently, Headway 17 and SL10 were guided to the left posterior cerebral artery and the second regrowth of the cerebral aneurysm, respectively (Figure 5c). A 3.5 mm × 23 mm LVIS Jr. (MicroVention, Terumo, Tustin, CA, USA) was deployed (Figure 5d). A 1.5 mm × 3 cm HyperSoft 3D was implanted through the jail lumen of the stent; then, the coil diameter was lowered and the coils were implanted. As a contrast-enhanced area was also observed between the initially treated and re-enlarged areas, another coil was placed in the same area to achieve embolization with six coils (Figure 5e).

The next day after surgery, a head MRI or MRA showed no obvious acute cerebral infarction or vascular occlusion, respectively. She was discharged from the hospital on day 6, June 19, 2021, with an mRS score of 0 without neurological deterioration. Cerebral angiography performed six months later showed no apparent recurrence, and MRA source images performed one year later showed no apparent recurrence.

## Discussion

SAH with abducens nerve palsy is reportedly found in 2.43% [1], 2.94% [2], and 5.9% [3] of SAH cases. Mano et al. [4] revealed that the anterior communicating artery was the most common site of hemorrhage (32.5%), followed by the VA (27.5%) and the internal carotid artery (20%), indicating that abducens nerve palsy can occur regardless of the site of hemorrhage. To the best of our knowledge, there are scarce reports of abducens nerve palsy associated with the rupture of a cerebral aneurysm at the tip of the basilar artery, and this is the first report of abducens nerve palsy occurring after each rupture.

In our case, we could not perform a detailed examination of eye movements at the time of the first and second SAHs because the light stimulation by opening the eyes may increase the risk of rerupture. So we performed a minimal examination, including pupillary irregularities and light reflexes. Therefore, on both occasions, the presence of right abducens nerve palsy became apparent upon awakening from postoperative anesthesia. Strictly speaking, the right abducens nerve palsy could have occurred at the time of disease onset or could have occurred due to transient ischemia caused by catheter manipulation. However, as the two postoperative MRI scans did not show any obvious ischemic lesions that could have caused the right abducens nerve palsy in each rupture and it was unlikely that the catheterization could have caused the right abducens nerve palsy on both occasions, we considered it more likely that the right abducens nerve palsy occurred at the time of disease onset.

The mechanism of abducens nerve palsy associated with SAH is believed to be direct compression by hematoma or contact with the VA system by hematoma or a jet of blood flow during rupture [3,5-7]. Munakata et al. [3] reported that abducens nerve palsy often occurs in patients with thick hematomas in the prepontine cistern. The vulnerability of the abducens nerve due to intracranial lesions has been reported previously, either because the abducens nerve runs the longest or because of its complex location with the cerebellar tent and blood vessels from the pontomedullary junction to Dorello’s canal, which may be affected by intracranial pressure fluctuations and contact with arteries [8]. In the present case, no obvious cerebral infarction or hemorrhage was detected in the brain stem on imaging that would have caused abducens nerve palsy after the onset of symptoms. The initial aneurysm exhibited an elongated shape at the tip of the basilar artery, and the second rupture occurred in the neck region of the regrowth of the initially treated aneurysm. Because the shape of the cerebral aneurysm differed between the first and second ruptures, the direction of the jet of blood flow during rupture may have been different. We hypothesized that the abducens nerve palsy was caused not by the jet of blood flow during rupture but rather by the location of the abducens nerve in relation to the surrounding structures, which were susceptible to the effects of hematoma or intracranial pressure fluctuations. Although the intracranial pressure fluctuations, in this case, are not known because we do not measure intracranial pressure routinely at our hospital, we may be able to infer intracranial pressure fluctuations as one of the hypotheses. A head CT revealed that the hematoma in the prepontine cistern was not markedly thicker at the time of disease onset, and it was unclear whether the hematoma thickness was affected. She also developed right-sided abducens nerve palsy on both occasions during the onset of SAH. We hypothesized that the right abducens nerve was more sensitive to the influence of the hematoma or intracranial pressure fluctuations than the left abducens nerve because of its position in relation to the surrounding structures.

In previous studies, several cases have reported improvement in SAH-associated abducens nerve palsy [4]. Laun et al. [1] reported that 50% of patients with SAH having abducens nerve palsy recovered within one week, 75% recovered within one month, and 89% showed full recovery [2]. In our case, the patient showed improvement on day 14 during the first event of rupture; however, during the second event of rupture, residual subjective symptoms of diplopia persisted until the time of hospital discharge on day 30, but her subjective symptoms completely resolved at the time of outpatient examination two months after SAH onset. The WFNS and Hunt & Kosnik grades on the second rupture were worse than those on the first rupture, and improvement in the right abducens nerve palsy required more time. The time to recovery of abducens nerve palsy may be associated with disease severity.

Coil embolization and clipping are surgical treatments for cerebral aneurysms at the tip of the basilar artery. The advantage of the former is minimally invasive, while the disadvantage is the possibility of recurrence due to coil compaction. The advantage of the latter is that the possibility of recurrence is low due to reliable clipping, but the disadvantage is that it is highly invasive. Nguyen et al. [9] reported that the intraoperative rupture rate of small cerebral aneurysms with a diameter of >3 mm (2.3%) is higher than that of aneurysms with a diameter of <3 mm (11.7%). Jindal et al. [10] performed coil embolization of microaneurysms with a diameter of <2 mm using Target Nano and revealed good results, regardless of whether the aneurysm had ruptured. In the present case, coil embolization was the treatment of choice because the hospital has an endovascular surgeon who can perform coil embolization promptly. The size of the cerebral aneurysm was approximately 2 mm at the time of initial treatment as well as regrowth, and the risk of intraoperative rupture caused by endovascular treatment was considered high. During the second regrowth, DAPT was initiated one week before treatment, and stent-assisted coil embolization was performed with a good outcome. Stent-assisted coil embolization of microaneurysms reportedly prevents recurrence without increasing the risk of complications [11]. The use of a stent allows the coil to be placed within the aneurysm and in the neck region of the aneurysm, increasing coil density and decreasing stent porosity in the neck region. The coil density can be increased, and the stent porosity in the neck region can be reduced. Stent wires crossing the neck region of the aneurysm have also been reported to provide a structural substrate for endothelialization, alleviate hemodynamic conditions, and enhance recurrence prevention. Stent-assisted coil embolization should be considered a treatment option for recurrent enlargement of cerebral aneurysms.

## Conclusions

We reported the case of a patient with a ruptured microaneurysm at the tip of the basilar artery who was presented with right abducens nerve palsy at the time of the initial rupture and rerupture during an outpatient follow-up. In the present case, the prognosis of SAH-associated abducens nerve palsy was good, and the time to improvement may depend on disease severity. Stent-assisted coil embolization is an effective treatment option for repeated regrowths after treatment of microaneurysms.
